# BxCDP1 from the pine wood nematode *Bursaphelenchus xylophilus* is recognized as a novel molecular pattern

**DOI:** 10.1111/mpp.12939

**Published:** 2020-04-21

**Authors:** Long‐Jiao Hu, Xiao‐Qin Wu, Hai‐Yang Li, Yuan‐Chao Wang, Xin Huang, Yan Wang, Yu Li

**Affiliations:** ^1^ Co‐Innovation Center for Sustainable Forestry in Southern China College of Forestry Nanjing Forestry University Nanjing China; ^2^ Jiangsu Key Laboratory for Prevention and Management of Invasive Species Nanjing Forestry University Nanjing China; ^3^ Department of Plant Pathology Nanjing Agricultural University Nanjing China

**Keywords:** *Bursaphelenchus xylophilus*, cell death, histological responses, innate immunity, molecular pattern, pathogenesis‐related genes

## Abstract

The migratory plant‐parasitic nematode *Bursaphelenchus xylophilus* is the causal agent of pine wilt disease, which causes serious damage to pine forests in China. Plant immunity plays an important role in plant resistance to multiple pathogens. Activation of the plant immune system is generally determined by immune receptors, including plant pattern recognition receptors, which mediate pattern recognition. However, little is known about molecular pattern recognition in the interaction between pines and *B. xylophilus*. Based on the *B. xylophilus* transcriptome at the early stages of infection and *Agrobacterium tumefaciens*‐mediated transient expression and infiltration of recombinant proteins produced by *Pichia pastoris* in many plant species, a novel molecular pattern (BxCDP1) was characterized in *B. xylophilus*. We found that *BxCDP1* was highly up‐regulated at the early infection stages of *B. xylophilus*, and was similar to a protein in *Pararhizobium haloflavum*. BxCDP1 triggered cell death in *Nicotiana benthamiana* when secreted into the apoplast, and this effect was dependent on brassinosteroid‐insensitive 1‐associated kinase 1, but independent of suppressor of BIR1‐1. BxCDP1 also exhibited cell death‐inducing activity in pine, *Arabidopsis*, tomato, pepper, and lettuce. BxCDP1 triggered reactive oxygen species production and the expression of PAMP‐triggered immunity marker genes (*NbAcre31*, *NbPTI5*, and *NbCyp71D20*) in *N. benthamiana*. It also induced the expression of pathogenesis‐related genes (*PtPR‐3*, *PtPR‐4*, and *PtPR‐5*) in *Pinus thunbergii*. These results suggest that as a new *B. xylophilus* molecular pattern, BxCDP1 can not only be recognized by many plant species, but also triggers innate immunity in *N. benthamiana* and defence responses of *P. thunbergii*.

## Graphical abstract

BxCDP1 can not only trigger innate immunity of the nonhost plant *Nicotiana benthamiana*, which depends on co‐receptors NbBAK1, but also induces defence responses of the host *Pinus thunbergii*.

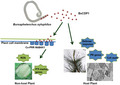

## 1 INTRODUCTION

The pine wood nematode (PWN) *Bursaphelenchus xylophilus* is an extremely damaging migratory plant‐parasitic nematode that infects most *Pinus* species, including *P. densiflora*, *P. massoniana*, *P. thunbergii*, and *P. pinaster*, resulting in massive economic losses in eastern Asia and Europe, especially in China and Japan (Hunt, 2009; Vicente *et al.*, 2011; Jones *et al.*, 2013; Zhou *et al.*, 2017; Li *et al.*, 2018b; Huang *et al.*, 2019). Pine wilt disease (PWD) caused by PWN is now considered the most serious threat to pine forests worldwide and is very difficult to control.

*B. xylophilus* exhibits phytophagous and mycophagous stages during feeding. At the phytophagous stage, the nematode migrates to the xylem resin and ray canals and feeds on parenchyma cells, leading to the death of these cells (Mamiya, 2012). As part of a strong defence response during the early stages of infection, the tree releases reactive oxygen species (ROS), polyphenolic compounds, terpenoids, and lipid peroxides (Fukuda, 1997). As the tree dies, the nematode switches to the mycophagous stage and feeds on the fungi that colonize the tree (Jones *et al.*, 2008).

Similar to most pathogens and parasites, *B. xylophilus* must overcome plant immunity to achieve successful host colonization. Typically, the plant innate immune system has two layers. Pathogen‐ or microbe‐associated molecular patterns (PAMPs or MAMPs, respectively), historically termed elicitors, are recognized by plant plasma membrane‐bound receptors (pattern recognition receptors [PRRs], including leucine‐rich repeat receptor‐like proteins [LRR‐RLPs] and leucine‐rich repeat receptor‐like kinases [LRR‐RLKs]) to induce the first tier of innate immunity (PAMP‐triggered immunity [PTI]) (Jones and Dangl, 2006). The LRR‐RLK brassinosteroid‐insensitive 1‐associated kinase 1 (BAK1) and suppressor of BIR1‐1 (SOBIR1) serve as coreceptors of multiple PRRs and participate in several types of PTI signalling pathways (Heese *et al.*, 2007; Liebrand *et al.*, 2014). The pathogen also secretes effectors to suppress PTI, facilitating infection. Recognition of some effectors, historically termed avirulence (Avr) proteins, by the nucleotide‐binding and leucine‐rich repeat (NB‐LRR) proteins encoded by disease resistance (R) genes triggers the second overlapping mode of innate immunity (effector‐triggered immunity [ETI]) (Chisholm *et al.*, 2006; Jones and Dangl, 2006). Plant innate immunity is characterized by accumulation of ROS, deposition of callose, and regulation of hormone signalling and induction of pathogenesis‐related (PR) genes (Dodds and Rathjen, 2010; Tsuda and Katagiri, 2010).

In addition to PAMPs and effectors that can trigger cell death and plant immunity, various cytolytic toxins produced by phytopathogenic microorganisms have also been shown to trigger plant immunity‐associated responses (van’t Slot *et al* *.*, 2002). These toxins include *Fusarium* spp.‐derived fumonisin B1, *Phomopsis amygdali*‐derived fusicoccin or *Cochliobolus victoriae*‐derived victorin (van’t Slot *et al* *.*, 2002; Qutob *et al* *.*, 2006). Toxin‐mediated host immune activation is likely to be the result of toxin‐mediated interference with host integrity (Ottmann *et al* *.*, 2009). For example, various necrosis and ethylene‐inducing peptide 1 (Nep1)‐like proteins (NLPs) produced by bacterial, oomycete, and fungal microbes are cytolytic toxins that trigger plant immunity‐associated defences by toxin‐induced host cell damage (plasma membrane destruction and cytolysis) (Ottmann *et al* *.*, 2009). A subsequent study further found that NLPs triggered plant immunity‐associated defences by two different mechanistic modes (i.e., toxin action and a classical PAMP motif [nlp20]) (Böhm *et al* *.*, 2014).

Plants recognize many different PAMPs from a range of plant pathogens. Bacterial PAMPs include flagellin and the flagellin‐derived peptides flg22 and flgII‐28 (Felix *et al.*, 1999; Gomez‐Gomez and Boller, 2000), elongation factor Tu (EF‐Tu) and the EF‐Tu‐derived peptides elf18 and elf26 (Kunze *et al.*, 2004; Zipfel *et al.*, 2006), and cold shock proteins (Felix and Boller, 2003). Among these PAMPs, flg22 is the best characterized. Several fungal PAMPs have been identified, such as chitin, *Rhynchosporium commune* RcCDI1, and *Valsa mali* VmE02 (Franco‐Orozco *et al.*, 2017; Nie *et al.*, 2018). Oomycete PAMPs include *Phytophthora infestans* INF1, β‐glucans, heptaglucoside, transglutaminase (Pep13), cellulose‐binding elicitor lectins, elicitins, and *Phytophthora sojae* glycoside hydrolase 12 protein (PsXEG1) (Ma *et al.*, 2015). In plant‐parasitic nematodes, ascaroside ascr18, a conserved nematode signalling molecule, elicits MAMP‐triggered immunity and pathogen resistance (Manosalva *et al.*, 2015). In addition, water in which invasive stage juvenile nematodes have been incubated (NemaWater) has been shown to elicit PTI responses in host plants (Mendy *et al.*, 2017, Wang'ombe, 2019). However, PAMPs have yet to be described from *B. xylphilus*, although several effectors have been characterized, including glycoside hydrolase family 45 (GH45) cellulases, pectate lyases, expansins, β‐1,3‐endoglucanases, and the novel effector BxSapB1 (Hu *et al.*, 2019).

In the present study, we selected 15 candidate effectors that were up‐regulated in the *B. xylophilus* transcriptome at the early stages of infection (Hu *et al.*, 2019). We then carried out transient expression in *Nicotiana benthamiana* using a potato virus X (PVX) expression vector and identified a *B. xylophilus* transcript (*BxCDP1*) that was highly abundant at the early stages of pine infection and that encoded a protein that triggered cell death in *N. benthamiana*. BxCDP1 was also able to induce cell death in pine and many other plants. Moreover, BxCDP1‐triggered cell death was dependent on NbBAK1 but did not require NbSOBIR1 and induced immune responses in *N. benthamiana*. BxCDP1 could also induce a defensive reaction in *P. thunbergii*. These data suggest that BxCDP1 may act as a novel molecular pattern in *B. xylophilus*. Furthermore, PhCDP1 from *Pararhizobium haloflavum*, a protein similar to BxCDP1, also induced cell death in *N. benthamiana*.

## 2 RESULTS

### 2.1 BxCDP1 induces cell death in *N. benthamiana*

**FIGURE 1:**
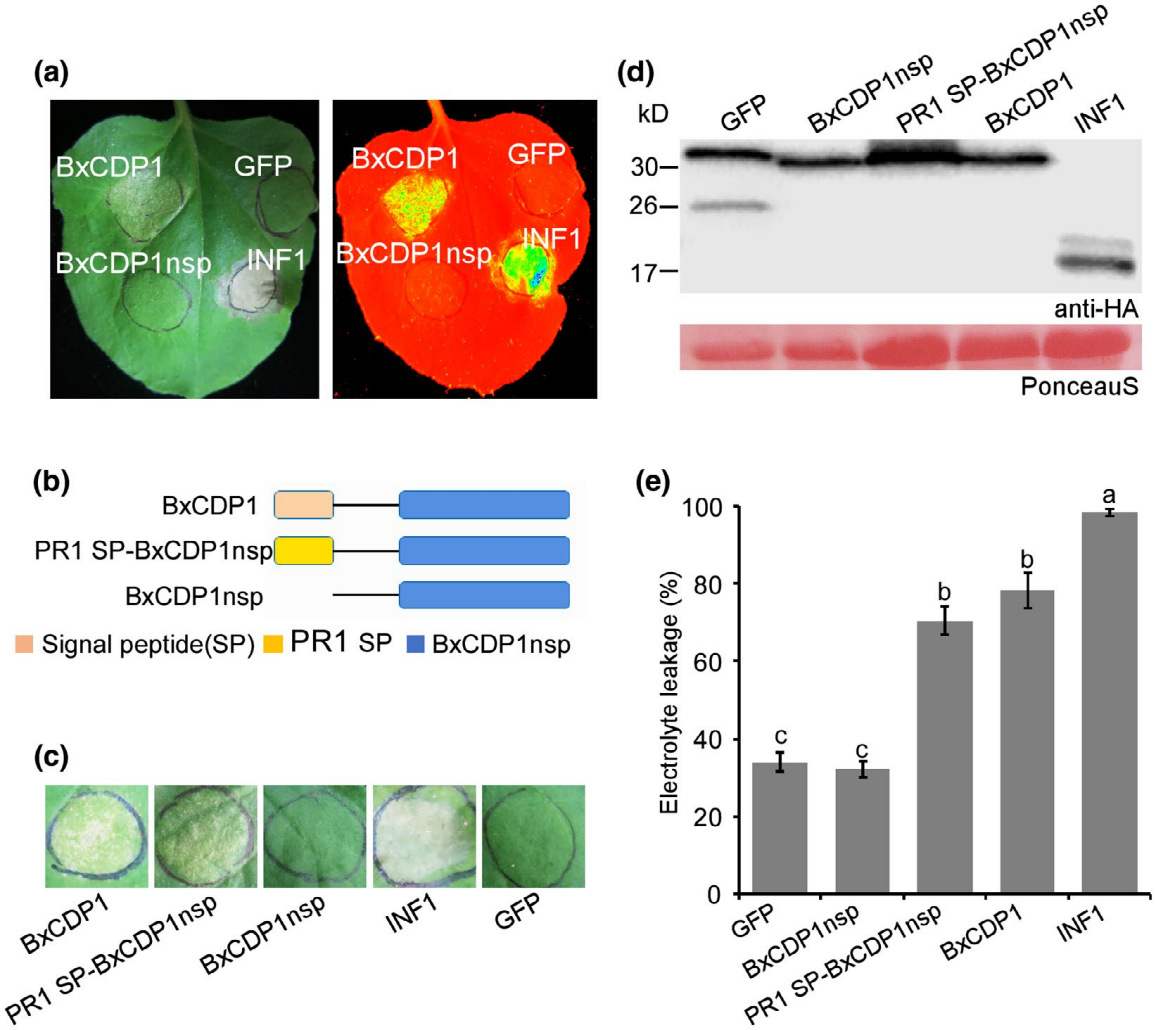
BxCDP1 triggers cell death in *Nicotiana benthamiana.* (a) Representative *N. benthamiana* leaves at 7 days after inoculation with *Agrobacterium tumefaciens* GV3101 carrying the *BxCDP1* gene in the vector pGR107. Representative *N. benthamiana* leaves were also placed under UV illumination to observe cell death. The infiltration assay was performed three times, and three different plants with three inoculated leaves were used in each assay. (b) Regions of BxCDP1 examined for cell death activity. (c) Representative *N. benthamiana* leaves at 7 days after agroinfiltration carrying BxCDP1nsp (no signal peptide) and PR1 SP‐BxCDP1nsp. The infiltration assay was performed three times, and three different plants with three inoculated leaves were used in each assay. (d) Immunoblot analysis of proteins from *N. benthamiana* leaves transiently expressing target proteins fused with 3 × HA tags. The experiment was repeated three times with similar results. (e) Quantification of cell death by measuring electrolyte leakage in *N. benthamiana* leaves at 7 days post‐infiltration with constructs encoding the indicated proteins. GFP, green fluorescent protein. The experiment was repeated three times with similar results. The data are the means, and the error bars represent ± *SD* from three independent experiments. Different letters on top of the bars indicate statistically significant differences (*p* < .05, *t* test), as measured by Duncan's multiple range test

Among the 247 up‐regulated genes from the *B. xylophilus* transcriptome (accession number: PRJNA397001) at the early stages of infection, we screened 69 candidate effectors (Hu *et al.*, 2019). Fifteen of the 69 candidate effectors with signal peptides were cloned separately into the binary PVX vector pGR107, which adds a C‐terminal 3 × hemagglutinin (HA) tag to the protein. The pGR107::*GFP* and pGR107::*INF1* constructs served as negative and positive controls, respectively. Transient expression of the genes in *N. benthamiana* demonstrated that BXY_1336500, with its endogenous signal peptide (denoted as BxCDP1 [cell death protein 1]) for secretion into the plant apoplast, induced strong cell death at 7 days after infiltration; in contrast, the truncated (lacking a signal peptide) version of BxCDP1 (BxCDP1nsp) did not induce cell death. Accordingly, the cell death regions triggered by BxCDP1 and the positive control INF1 emitted an intense fluorescence signal under UV illumination, but the regions infiltrated with BxCDP1nsp and the negative control green fluorescent protein (GFP) were not fluorescent (Figure 1a). The Simple Modular Architecture Research Tool (SMART) identified no protein domains in this protein of unknown function. Its open reading frame (ORF) is 708 bp and encodes a 236‐amino acid polypeptide that contains three cysteine residues. We carried out further studies on this protein.

To determine whether apoplastic localization is required for BxCDP1‐triggered cell death, the signal peptide from PR protein 1 (PR1) of *Nicotiana tabacum* (Table S2) was added to the N‐terminus of BxCDP1nsp to produce PR1 SP‐BxCDP1nsp (Figure 1b). Transient assays showed that BxCDP1 and PR1 SP‐BxCDP1nsp, but not BxCDP1nsp, triggered cell death in *N. benthamiana* (Figure 1c), and the expression of these proteins was validated by western blot analysis (Figure 1d). Ion leakage measurements were then employed to quantify the degree of cell death triggered by the five tested proteins. The electrolyte leakage induced by INF1, BxCDP1, and PR1 SP‐BxCDP1nsp was significantly higher than that induced by BxCDP1nsp or GFP (Figure 1e). These data indicate that BxCDP1 triggers cell death in *N. benthamiana* when secreted into the apoplast.

### 2.2 *Pararhizobium haloflavum *contains a similar protein to BxCDP1 that also induces cell death in *N. benthamiana*

Querying the BxCDP1 amino acid sequence against the National Center for Biotechnology Information (NCBI) protein database resulted in the identification of only one similar protein, which was present in *P. haloflavum* (denoted as PhCDP1), recently identified as a novel species of *Pararhizobium* (Shen *et al.*, 2018). The nucleotide and amino acid sequences of BxCDP1 share 87% and 77% identity, respectively, with that of PhCDP1 (Figure S1a,b). In order to check that the *B. xylophilus* gene was of nematode origin, it was cloned from genomic DNA of *B. xylophilus* (Figure S4a,b). In addition, the sequence was identified in the genome assembly for *B. xylophilus* and was also present in a sequence present in a transcriptome analysis of this species (Kikuchi *et al.*, 2011; Tsai *et al.*, 2016).

To determine whether PhCDP1 also induces cell death in *N. benthamiana*, *PhCDP1* was cloned separately into the pBINRFP vector, which adds a C‐terminal red fluorescent protein (RFP) tag to the protein. Then, the protein was transiently expressed in *N. benthamiana*. The results show that PhCDP1 also triggered cell death in *N. benthamiana* (Figure S1c). The expression of PhCDP1 was validated by western blot analysis (Figure S1d).

### 2.3 BxCDP1 induces cell death in *N. benthamiana*, *Arabidopsis*, tomato, pepper, and lettuce

To further confirm that the BxCDP1 protein is able to induce cell death in *N. benthamiana*, BxCDP1 was produced in the yeast *Pichia pastoris* using the pPICZαA vector (pPICZαA:BxCDP1), which contains a 6 × His tag and targets the protein for secretion into the culture medium. As described in previous studies (Ma *et al* *.*, 2015; Wang *et al.*, 2018), *P. pastoris* culture supernatant from the pPICZαA empty vector (EV) control strain was used as the control in this assay and was purified in the same way as BxCDP1. After recovery from the culture supernatant using Ni‐NTA resin, the recombinant protein BxCDP1rec and EV were detected by SDS‐polyacrylamide gel electrophoresis (SDS‐PAGE) and western blot analysis, respectively. The results showed that BxCDP1 was successfully induced and purified (Figure S2).

**FIGURE 2:**
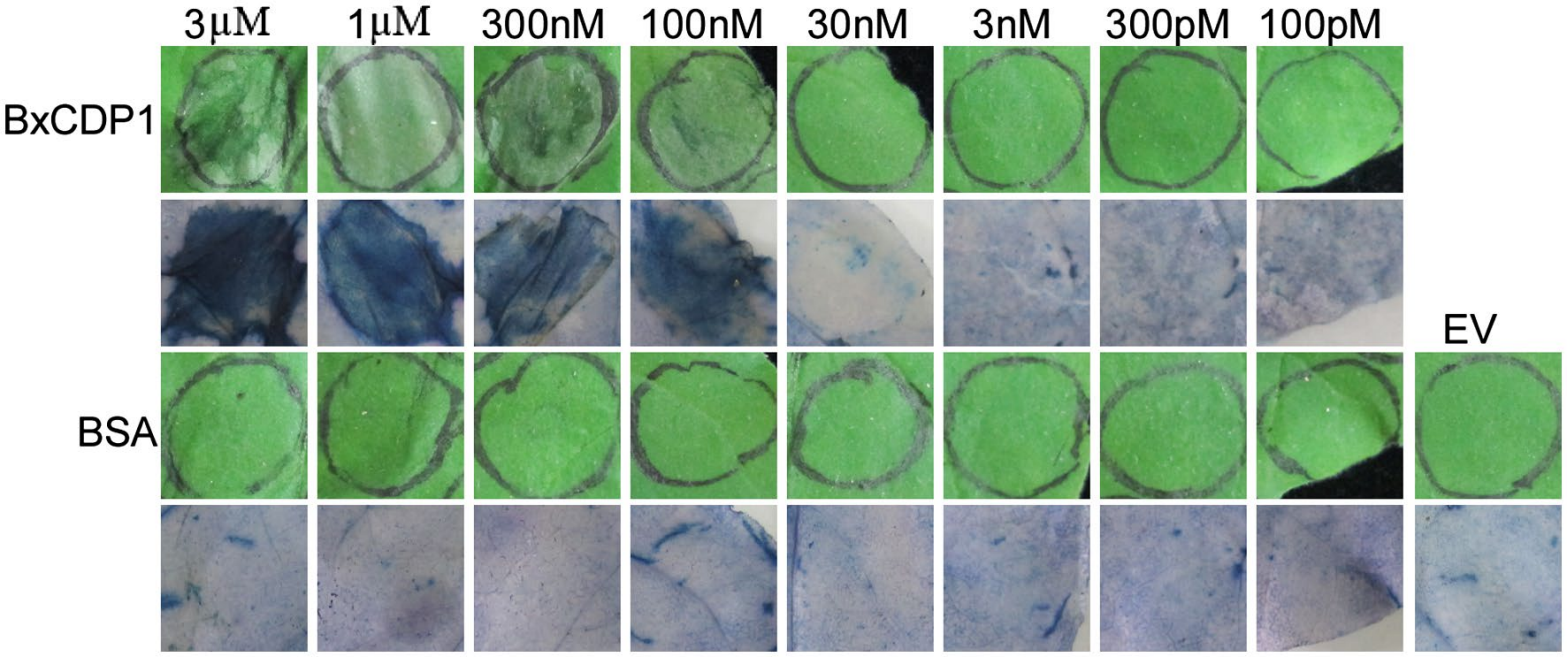
Representative *Nicotiana benthamiana* leaves infiltrated with the purified BxCDP1 protein. Bovine serum albumin (BSA) and empty vector (EV) were used as controls. The infiltration assay was repeated three times with similar results, and three different plants with three inoculated leaves were used in each assay

BxCDP1rec was tested for cell death activity by infiltrating 100 pM to 3 µM protein solution into the mesophyll of *N. benthamiana* leaves. EV and bovine serum albumin (BSA) were used as controls. In this system, BxCDP1rec induced cell death at 3 days after infiltration (Figure 2). Moreover, cell death was enhanced with increasing concentrations of BxCDP1 from 100 pM to 3 µM.

**FIGURE 3:**
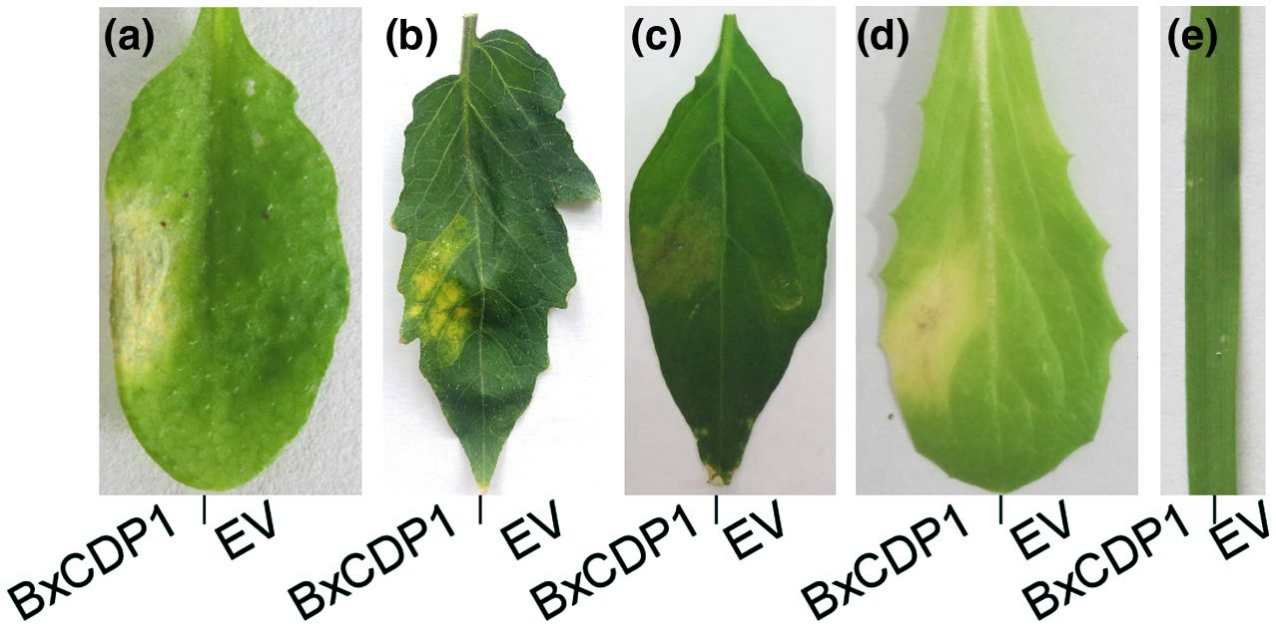
Cell death response in diverse species of plants triggered by the purified BxCDP1 protein. (a) and (b) Representative *Arabidopsis* and tomato leaves at 5 days after inoculation with 300 nM BxCDP1 protein. (c) Representative pepper leaves at 7 days after inoculation with 300 nM BxCDP1 protein. (d) and (e) Representative lettuce and wheat leaves at 7 days after inoculation with 1 µM BxCDP1 protein. Empty vector (EV) was used as a control. These infiltration assays were repeated three times with similar results, and three different plants with three inoculated leaves were used in each assay

To examine the host specificity of BxCDP1, we infiltrated the purified protein into expanded leaves of various plant species. EV was used as the control. BxCDP1 at 300 nM induced cell death in *Arabidopsis thaliana*, *Solanum lycopersicum*, and *Capsicum annuum*, and 1 µM BxCDP1 induced cell death in *Lactuca sativa* var. *romana* (Figure 3a–d), but not in *Triticum aestivum* (Figure 3e). The result was consistent with the agroinfiltration results (Figure S3). Thus, BxCDP1 can induce cell death in diverse plant families.

### 2.4 NbBAK1, but not NbSOBIR1, is required for BxCDP1‐triggered cell death in *N. benthamiana*

**FIGURE 4:**
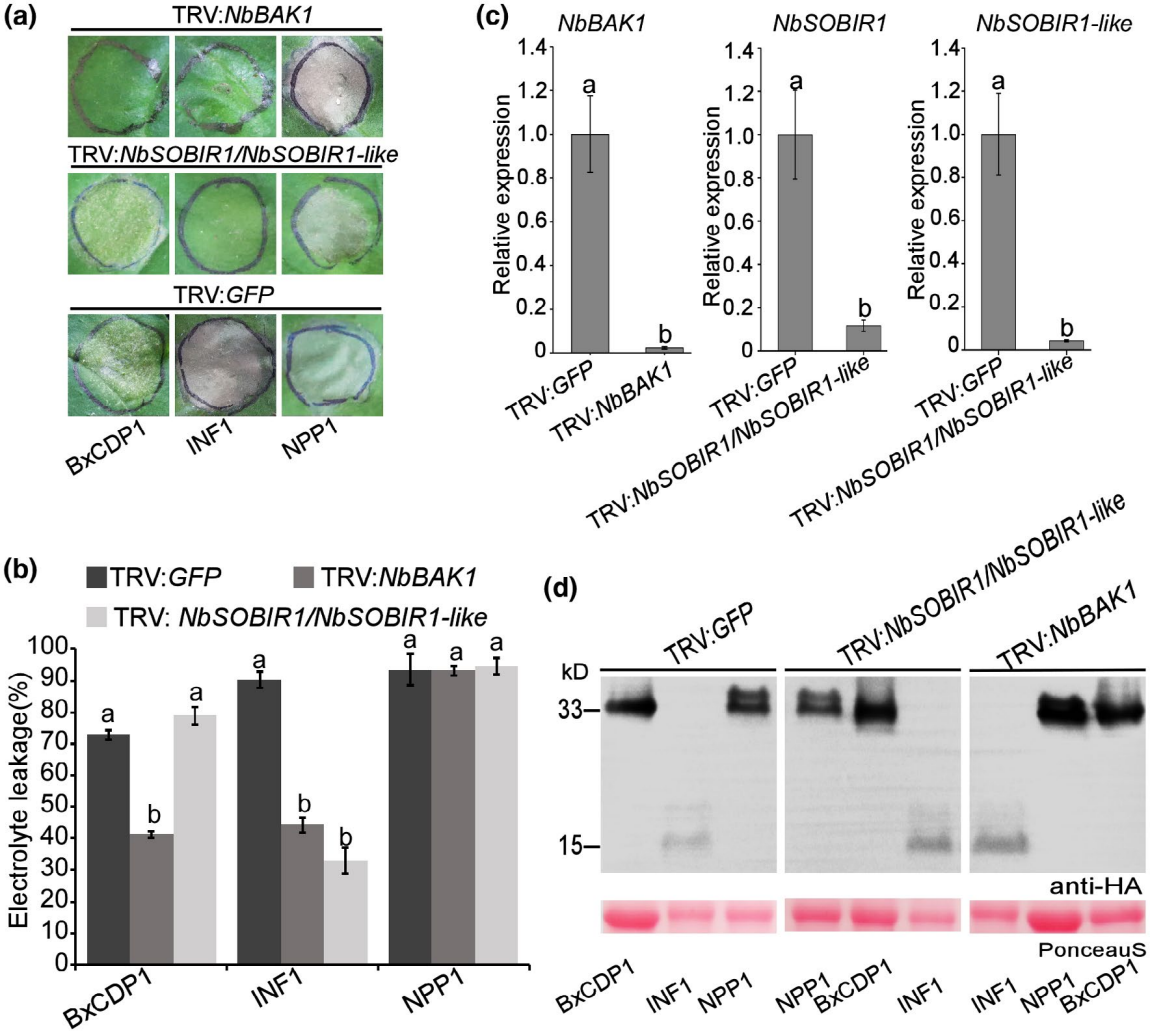
Cell death triggered by BxCDP1 requires *Nicotiana benthamiana* brassinosteroid insensitive 1‐associated kinase 1 (NbBAK1) but not *N. benthamiana* suppressor of BIR1‐1 (NbSOBIR1). (a) Representative photographs of agroinfiltration in *NbBAK1*‐, *NbSOBIR1*‐, and *GFP*‐silenced plants. The infiltration assay was repeated three times, and three different plants with three inoculated leaves were used in each assay. (b) Quantification of cell death by measuring electrolyte leakage in *N. benthamiana* leaves at 7 days post‐infiltration with constructs encoding the indicated proteins. The experiment was repeated three times with similar results. (c) Silencing efficiencies of *NbBAK1*, *NbSOBIR1*, and the *NbSOBIR1* homolog *NbSOBIR1‐like* after virus‐induced gene silencing (VIGS) treatment, as determined by quantitative reverse transcription PCR (RT‐qPCR) analysis. Data are the means, and the error bars represent ± *SD* from three biological replicates. Different letters on top of the bars indicate statistically significant differences (*p* < .05, *t* test) as measured by Duncan's multiple range test. (d) Western blot of proteins from *N. benthamiana* leaves transiently expressing BxCDP1, INF1, and NPP1 fused with 3 × HA. The experiment was repeated three times with similar results

As coreceptors for different PRRs, BAK1 and SOBIR1 participate in multiple PRR pathways, including cell death induction by the *P. infestans* PAMP INF1 and the *Rhynchosporium commune* PAMP RcCDI1 (Franco‐Orozco *et al* *.*, 2017). To determine whether NbBAK1 and NbSOBIR1 participate in the induction of cell death by BxCDP1, virus‐induced gene silencing (VIGS) constructs were used to independently target *NbBAK1* and *NbSOBIR1* expression in *N. benthamiana*. Because a previous study showed that necrosis‐inducing *Phytophthora* protein 1 (NPP1)‐triggered cell death did not depend on BAK1 and SOBIR1 (Wang *et al.*, 2018), these factors were used as controls. Three weeks after infiltration with the VIGS constructs, plants were agroinfiltrated with BxCDP1, INF1, and NPP1. As expected, INF1 induced cell death in *GFP*‐silenced plants but not in *NbBAK1‐* or *NbSOBIR1*‐silenced plants. NPP1 triggered cell death in all plants silenced with the three VIGS constructs. However, BxCDP1 triggered cell death in only *GFP‐* and *NbSOBIR1*‐silenced plants and not in *NbBAK1*‐silenced plants (Figure 4a). Furthermore, the degree of cell death triggered by these three proteins was measured, with BxCDP1‐triggered cell death being significantly reduced in *NbBAK1*‐silenced plants (Figure 4b).

The silencing constructs we used (TRV:*NbBAK1* and TRV:*NbSOBIR1*/*NbSOBIR1‐like*) were the same as previously used in similar studies (Liebrand *et al*, 2013; Wang *et al* *.*, 2018), which showed that *NbBAK1* and its homologs *NbBAK1a* and *NbBAK1b*, and *NbSOBIR1* and its homolog *NbSOBIR1‐like* were all silenced (Liebrand *et al*, 2013; Wang *et al* *.*, 2018). The silenced parts of the receptors (*NbBAK1* and *NbSOBIR1*) are shown in Table S3. The nucleic acid sequences of *NbBAK1a* and *NbBAK1b* are too similar to design specific primers to detect their individual expression. Thus, we detected the relative expression of *NbBAK1*, *NbSOBIR1*, and *NbSOBIR1‐like* by quantitative reverse transcription PCR (RT‐qPCR). The results confirmed that *NbBAK1*, *NbSOBIR1*, and *NbSOBIR1‐like* were all markedly silenced by the VIGS constructs (Figure 4c). According to the principle of VIGS, the *NbBAK1* dsRNA can be degraded by Dicer enzyme into siRNA of 21–23 nucleotides. Thus, the TRV:*NbBAK1* could also silence *NbBAK1a* and *NbBAK1b*. The nucleic acid sequences of *NbBAK1a*, *NbBAK1b*, and *NbSOBIR1‐like* are provided in Table S4. Moreover, western blotting verified that all assayed proteins were expressed in the silenced *N. benthamiana* plants (Figure 4d). Taken together, these results suggest that *NbBAK1* participated in BxCDP1‐triggered cell death but that *NbSOBIR1* did not.

### 2.5 BxCDP1 induces immune responses in *N. benthamiana*

**FIGURE 5:**
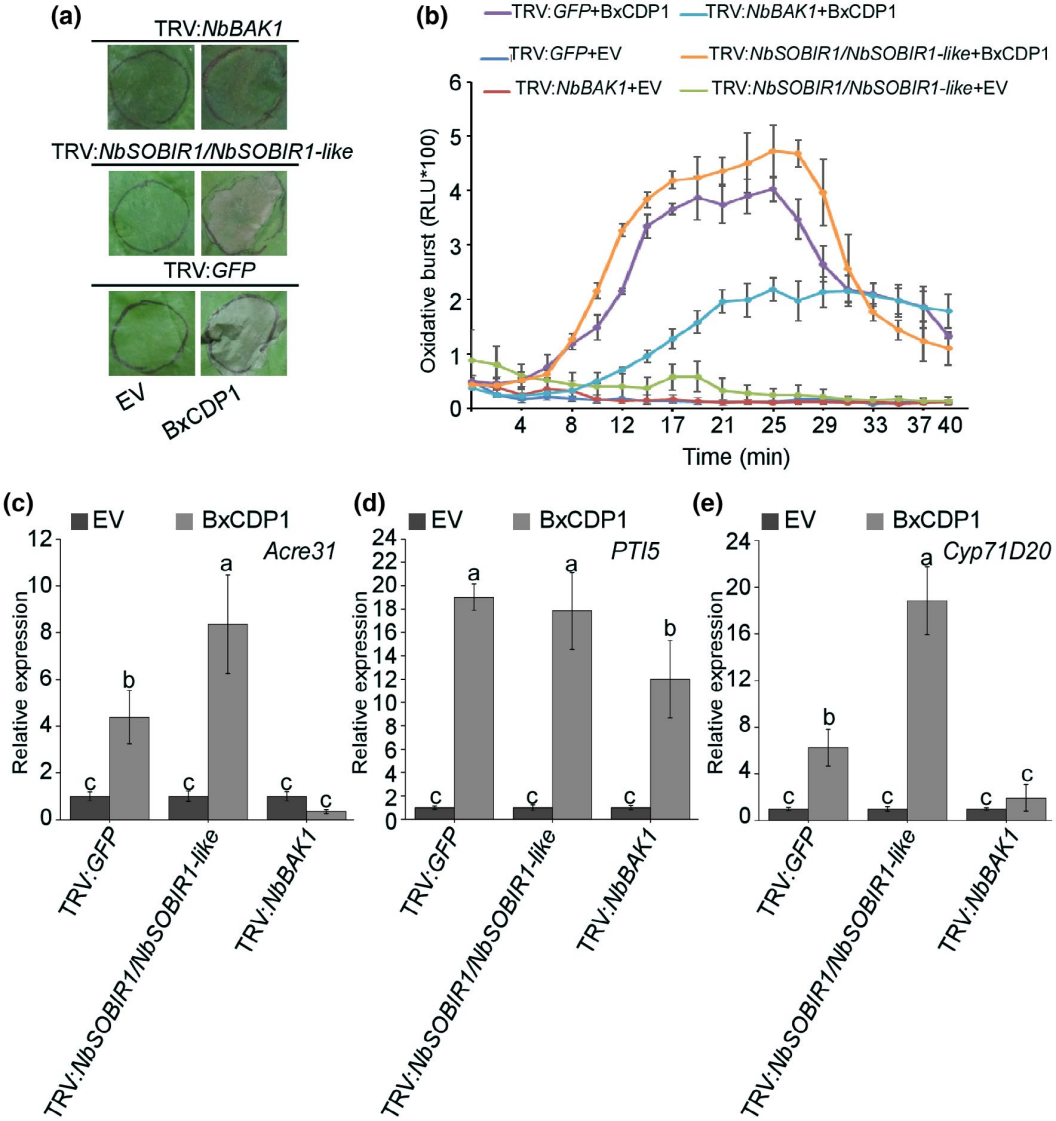
BxCDP1 induces immune responses in *Nicotiana benthamiana*. (a) Representative leaves showing cell death induced by expression of the purified BxCDP1 protein, empty vector (EV) solution, INF1, or NPP1 in *N. benthamiana* leaves treated with tobacco rattle virus:green fluorescent protein (TRV:*GFP*), TRV:*NbBAK1*, or TRV:*NbSOBIR1/NbSOBIR1‐like*. Infiltrated leaves were photographed at 3 days post‐infiltration. The infiltration assay was repeated three times, and three different plants with three inoculated leaves were used in each assay. (b) Reactive oxygen species (ROS) production in *NbBAK1*‐, *NbSOBIR1*‐, and *GFP*‐silenced *N. benthamiana* leaves treated with 1 μM BxCDP1 or EV. Experiments were repeated twice with similar results. (c)–(e) Transcriptional up‐regulation of *N. benthamiana* PAMP‐triggered immunity (PTI) marker genes triggered by 300 nM BxCDP1 was prevented in brassinosteroid insensitive 1‐associated kinase 1 (*BAK1*)‐silenced *N. benthamiana.* EV was used as a control. Data are the means, and the error bars represent ± *SD* from three biological replicates. Different letters on top of the bars indicate statistically significant differences (*p* < .05, *t* test) as measured by Duncan's multiple range test

To further determine whether BxCDP1 induces cell death, the purified BxCDP1 protein (300 nM) and EV were infiltrated into *GFP‐*, *NbBAK1‐*, and *NbSOBIR*1‐silenced plants. At 3 days after infiltration, the purified BxCDP1 protein triggered cell death in the *GFP‐* and *NbSOBIR1*‐silenced plants but not in the *NbBAK1*‐silenced plants (Figure 5a), consistent with the results of BxCDP1 infiltration. At the same time, the production of ROS triggered by the purified BxCDP1 protein in the *GFP‐* and *NbSOBIR1*‐silenced plants was significantly higher than that in the *NbBAK1*‐silenced plants. Conversely, EV scarcely triggered ROS production (Figure 5b). RT‐qPCR analysis was also conducted on a range of PTI marker genes in *N. benthamiana* leaves at 3 hr post‐infiltration with the purified BxCDP1 protein (300 nM) and EV. These results showed that *NbAcre31*, *NbPTI5*, and *NbCyp71D20* were significantly induced by BxCDP1 in *GFP‐* and *NbSOBIR*1‐silenced plants at levels much higher than the expression levels in *NbBAK1*‐silenced plants (Figure 5c–e). These data further show that BxCDP1 can induce immune responses in *N. benthamiana* and that NbBAK1 participates in the signalling pathways of immune responses triggered by BxCDP1.

### 2.6 *BxCDP1* is highly induced at early infection stages

**FIGURE 6:**
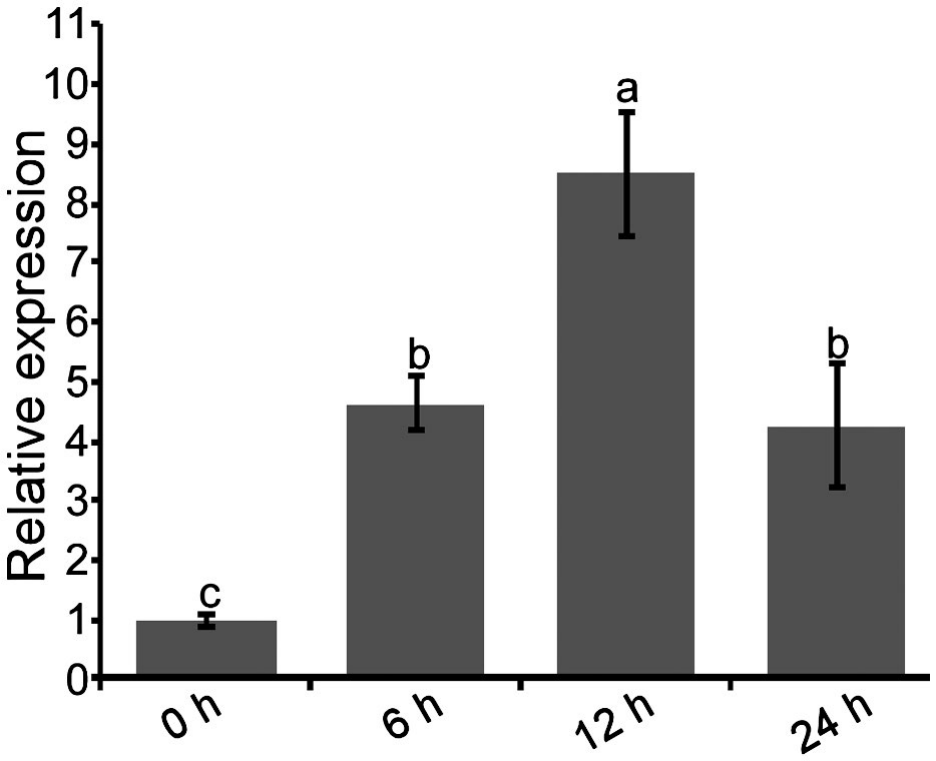
The expression pattern of BxCDP1 at the early stages of *Bursaphelenchus xylophilu*s infection by RT‐qPCR analysis. Relative expression of *BxCDP1* at the mycetophagous stage (0 hr) and at the early stages of infection (6, 12, and 24 hr). Data are the means, and the error bars represent ± *SD* from three biological replicates. Different letters on top of the bars indicate statistically significant differences (*p* < .05, *t* test) as measured by Duncan's multiple range test

According to our previous transcriptome data for *B. xylophilus*, BxCDP1 is up‐regulated at the early infection stages (Hu *et al.*, 2019). To further confirm this finding, RT‐qPCR was employed to obtain the expression profile of *BxCDP1* at the early stages of infection. The results showed that *BxCDP1* was indeed up‐regulated at early infection stages compared with the mycophagous stage (Figure 6), suggesting that *BxCDP1* plays an important role in the early stages of *B. xylophilus* infection.

### 2.7 BxCDP1 induces pathogenesis‐related marker genes expression of *P. thunbergii*

**FIGURE 7:**
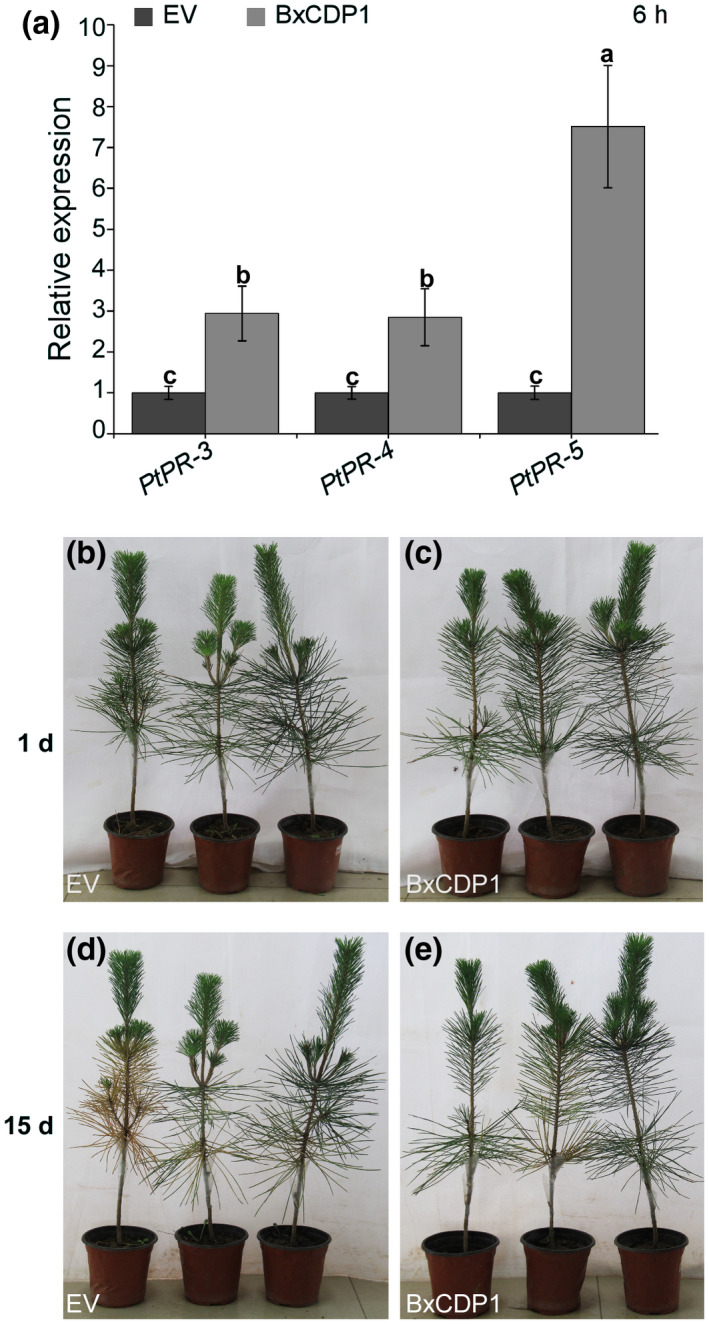
BxCDP1 induces pathogenesis‐related marker genes expression of *Pinus thunbergii*. (a) Relative transcript levels of pathogenesis‐related genes in *P. thunbergii* infected with the purified BxCDP1 protein and empty vector (EV). Stems c.2 cm in length were used for RNA extraction at 6 hr post‐inoculation. The inoculation assay was repeated three times, and in each assay three different seedlings for each treatment were used. Data are the means, and the error bars represent ± *SD* from three independent experiments. Different letters on top of the bars indicate statistically significant differences (*p* < .05, *t* test) as measured by Duncan's multiple range test. (b) and (c) Representative photographs of *P. thunbergii* at 1 day post‐inoculation. (d) and (e) Representative photographs of *P. thunbergii* at 15 days post‐inoculation. The infection assay was repeated three times

Plants launch defence responses when attacked by pathogens, and one of these defence responses is the induction of PR proteins (van Loon *et al.*, 2006). BxCDP1 was able to induce cell death in the nonhost plants tobacco, *Arabidopsis*, tomato, pepper, and lettuce and triggered PTI marker gene expression in *N. benthamiana*. However, it is unclear whether BxCDP1 induces a defence response and cell death in the host. Thus, we detected the relative expression of PR genes (*PtPR‐3*, *PtPR‐4*, and *PtPR‐5*) at 6 hr post‐inoculation by RT‐qPCR and found that *PtPR‐3*, *PtPR‐4*, and *PtPR‐5* were significantly up‐regulated in *P. thunbergii* compared with the expression levels in EV‐treated samples (Figure 7a).

Approximately 2,000 nematodes (a mixture of juveniles and adults) were inoculated into *P. thunbergii* seedlings, which were inoculated with purified BxCDP1 protein and EV for 4 hr. The results show that the early symptoms occurred later and the degree of morbidity of *P. thunbergii* seedlings inoculated with the former were significantly lower than those of seedlings inoculated with the latter (Figure 7b–e) at 15 days post‐inoculation.

### 2.8 BxCDP1 induces cell death in the host

**FIGURE 8:**
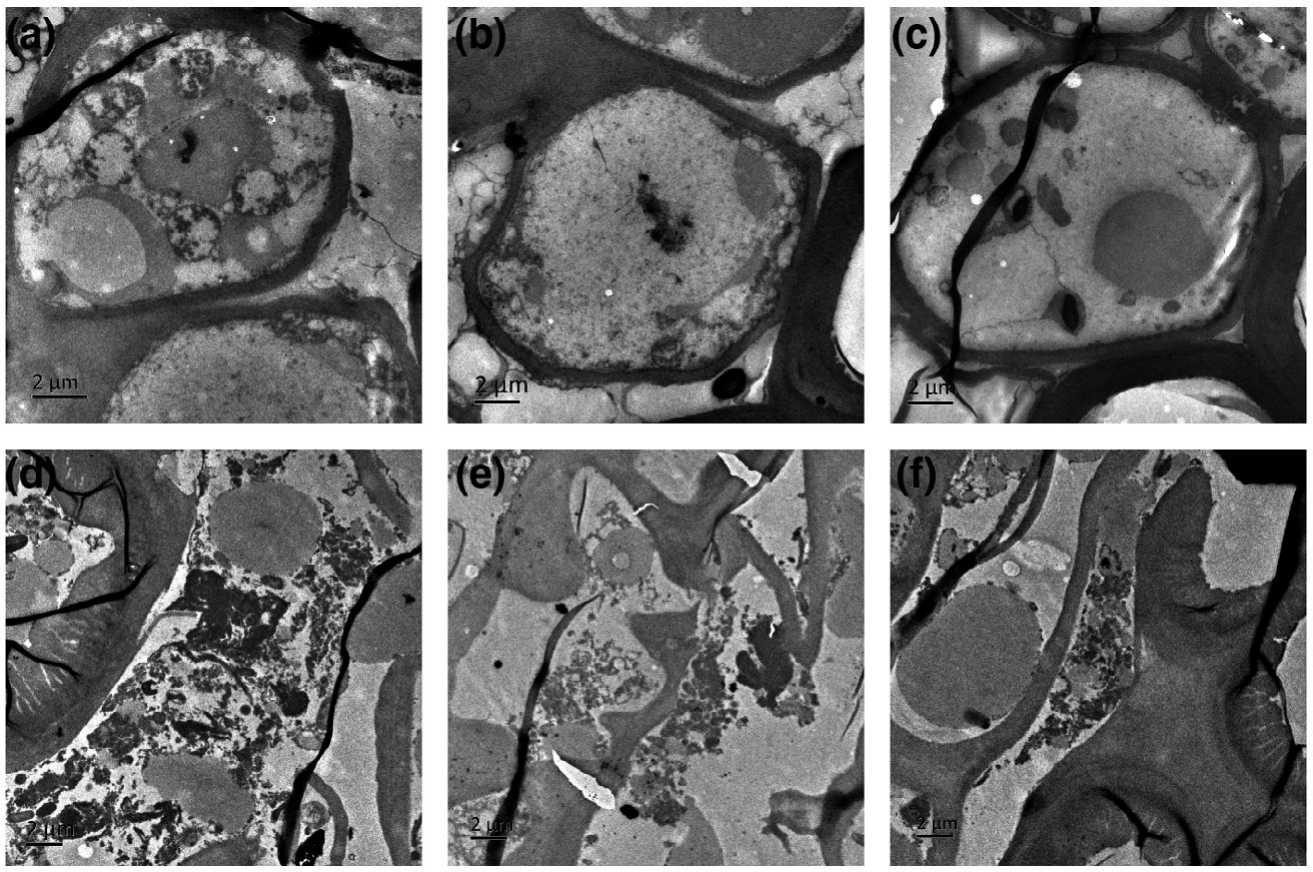
BxCDP1 induces cell death in pine. (a)–(c) Representative photographs of the cell morphology of *Pinus thunbergii* treated with empty vector (EV), as assessed by transmission electron microscopy (TEM). (d)–(f) Representative photographs of the cell morphology of *P. thunbergii* treated with the purified BxCDP1 protein, as assessed by TEM

As a gymnospermous plant, pine is obviously different from tobacco in many ways, and *A. tumefaciens* infiltration is not a suitable method of transformation for pine needles. Thus, the cell morphology of *P. thunbergii* was observed using transmission electron microscopy (TEM) at 10 days post‐inoculation. The *P. thunbergii* cells inoculated with EV exhibited morphological integrity (Figure 8a–c). In contrast, inoculation of *P. thunbergii* with the purified BxCDP1 protein led to cell lysis (Figure 8d–f). This result indicates that BxCDP1 induced cell death in the host, as it does in the model plant *N. benthamiana*.

## 3 DISCUSSION

We previously predicted 69 candidate effectors based on transcriptome analysis of *B. xylophilus* at the early stages of infection (Hu *et al.*, 2019). In the present study, we carried out transient expression of 15 of these candidate effectors in the model plant *N. benthamiana* for functional analysis. A protein named BxCDP1, containing three cysteine residues and a predicted signal peptide, was identified as triggering cell death in *N. benthamiana*. Because BxCDP1 is transcribed at a higher level at the early infection stages than at the mycetophagous stage, it may play an important role in the interaction with pine. Unfortunately, previously characterized protein domains that might provide clues about the possible function of this protein were not identified.

A number of studies have demonstrated that most known PRRs require BAK1 for their function (Heese *et al* *.*, 2007). In this study, BxCDP1‐triggered cell death was dependent on NbBAK1. Thus, the result suggests that BxCDP1 is not a general toxin and its detection might be mediated by a cell surface PRR. BxCDP1 triggers the transcriptional up‐regulation of several PTI marker genes in *N. benthamiana*, including *NbAcre31*, *NbPTI5*, and *NbCyp71D20*. However, silencing of *NbBAK1* significantly decreased this up‐regulation, further confirming the involvement of *NbBAK1* in BxCDP1 recognition. NbSOBIR1 was not required for BxCDP1 recognition, which suggests that the receptor detecting BxCDP1 in *N. benthamiana* is not an LRR‐RLP. Moreover, BxCDP1 can trigger cell death in the host plant pine and induce cell death in diverse plants such as tobacco, *Arabidopsis*, tomato, pepper, and lettuce. The results indicate that the cell death that occurred in these plants was induced through the same signalling pathway. Taken together, these data indicate that BxCDP1 may be a PAMP.

Typically, PAMPs are highly conserved within a class of microbes. For example, homologs of the oomycete PAMP PsXEG1 are widely present in prokaryotic and eukaryotic microorganisms, especially in plant‐associated microbes (oomycetes, fungi, and bacteria) (Ma *et al* *.*, 2015). In addition, homologs of the *R. commune* PAMP RcCDI1 are distributed widely across ascomycetes (Franco‐Orozco *et al* *.*, 2017). However, there are also some PAMPs that are less conserved (Brunner *et al* *.*, 2002; Lee *et al* *.*, 2009). For example, Pep‐13, a surface‐exposed fragment of a calcium‐dependent cell wall transglutaminase (TGase) that is conserved among *Phytophthora* species, can activate various plant defences (Brunner *et al* *.*, 2002). In this study, we attempted to match the BxCDP1 amino acid sequence with sequences in the NCBI protein database using BLASTP to identify homologs of BxCDP1, but only one similar protein, denoted as PhCDP1, was identified, which is from a bacterium (*P. haloflavum*). It showed that the nucleotide and amino acid sequences of BxCDP1 shared 87% and 77% identity, respectively, with the sequences of PhCDP1. Moreover, the nucleotide sequence of BxCDP1 was queried against genomes of all other nematodes in the WormBase ParaSite database and the transcriptome of *B. mucronatus* in NCBI, yet no homolog was retrieved. We undertook extensive analysis to confirm that this gene is derived from the nematode rather than from a bacterial contaminant. These analyses indicate that BxCDP1 is only present in *B. xylophilus* but not in other nematodes and is most similar to a bacterial sequence.

In this study, BxCDP1 induced the expression of some PR genes (*PtPR‐3*, *PtPR‐4*, and *PtPR‐5*) in *P. thunbergii.* Moreover, compared with the control, the onset of purified BxCDP1 protein in pines was delayed after inoculation with *B. xylophilus*. This finding indicates that BxCDP1 might trigger the defence response of the pines. We speculate that the enhancement of the defence response of the pines might further prevent *B. xylophilus* infection to some degree such that the onset of pine wilt was delayed.

In conclusion, BxCDP1 is a new molecular pattern that is recognized by the host pine. This study represents an important step toward understanding the interaction mechanisms between *B. xylophilus* and pine.

## 4 EXPERIMENTAL PROCEDURES

### 4.1 Nematodes and plant materials

A highly virulent strain of *B. xylophilus*, AMA3, was transferred into a mycelial mat of *Botrytis cinerea* growing on potato dextrose agar (PDA) plates and cultured at 25 °C for 7 days. The nematodes were extracted using the Baermann funnel technique (Qiu *et al.*, 2013). *N. benthamiana*, *A. thaliana*, *S. lycopersicum*, *C. annuum*, and *L. sativa* var. *romana* were grown in a glasshouse at 25 °C with a relative humidity of 60% under 16:8‐hr light:dark conditions. *P. thunbergii* (3‐year‐old) seedlings were cultivated at temperatures ranging from 28 to 32 °C with relative humidity ranging from 65% to 75%.

### 4.2 RNA isolation and cDNA synthesis

A suspension of approximately 10,000 mixed‐life‐stage nematodes was collected from PDA plates and inoculated onto *P. thunbergii* (3‐year‐old) seedlings for 6, 12, and 24 hr. The whole seedlings were cut into approximately 2‐cm segments of stems, which were split into halves. The nematodes were extracted from these segments of stems using a Baermann funnel. Total RNA from the nematodes was extracted using TRIzol reagent (Invitrogen). Leaves of *N. benthamiana* and stems of *P. thunbergii* were sampled and frozen in liquid nitrogen. Total RNA was extracted using the Plant Total RNA Kit (Zoman). First‐strand cDNA for RT‐qPCR was synthesized from 1 μg of total RNA using HiScript II Q RT SuperMix for qPCR (+gDNA wiper) (Vazyme) according to the manufacturer's protocol.

### 4.3 Plasmid constructs

The transcriptome of *B. xylophilus* at the early stages of infection (6, 12, and 24 hr post‐inoculation) was obtained in our previous study, with 69 candidate effectors (Hu *et al.*, 2019). Fifteen randomly selected candidate effectors with highly abundant expression were cloned using *B. xylophilus* cDNA and matched against the SWISS‐PROT database using BLAST (Table S1). The gene encoding *PhCDP1* was synthetized (GenScript). These fragments of the 15 candidate effectors were ligated into PVX (pGR107‐3 × HA), and *BxCDP1* and *PhCDP1* were ligated into pBINRFP (pCAM1300‐RFP) using the Clone Express II One Step Cloning Kit (Vazyme). Individual colonies for each construct were examined by PCR for insertions, and the selected clones were verified by sequencing. The primers used are listed in Table S1.

### 4.4 Expression and purification of the recombinant BxCDP1 protein

The ORF for BxCDP1 was amplified from *B. xylophilus* cDNA using primers with vector‐specific extensions (Table S1). The purified PCR product was inserted into the linearized vector pPICZαA (Invitrogen), which contains a 6 × His tag. The construct was transformed into *Escherichia coli* TOP10 competent cells. Individual colonies from the construct were tested by PCR for insertions, and the selected clones were verified by sequencing. The constructed plasmid was linearized with *Sac*I (New England Biolabs). The pPICZαA vector containing BxCDP1‐6 × His and EV were transformed into the *P. pastoris* KM71H (Invitrogen) via electroporation. Positive clones were grown in yeast extract‐peptone‐dextrose (YPD) medium containing 100 µg/ml zeocin at 30 °C for 3 days. BMGY (buffered glycerol‐complex medium) and BMMY (buffered methanol‐complex medium) were used for protein expression. After 4 days, the cultures were centrifuged at 6,500 rpm for 10 min to procure the supernatant containing BxCDP1‐6 × His and EV, respectively, as confirmed by SDS‐PAGE. Purification of the recombinant protein BxCDP1‐6 × His and EV from the culture supernatant was performed by affinity chromatography using Ni‐NTA Superflow resin (Qiagen).

### 4.5 *A. tumefaciens* and protein infiltration assays

Infiltration assays were performed according to a previous report (Hu *et al.*, 2019). In brief, constructs were transformed into *A. tumefaciens* GV3101 by electroporation, and the cells were grown on Luria Bertani agar plates with kanamycin and rifampicin. For agroinfiltration assays, recombinant *A. tumefaciens* strains were grown at 30 °C in a shaking incubator at a rotation speed of 200 rpm for 12 hr. For each construct, the bacterial cells were collected, resuspended in wash buffer (10 mM MgCl_2_, 10 mM  2‐(*N*‐morpholino)ethanesulfonic acid [MES], 100 µM acetosyringone [AS], pH 5.6) and diluted to a final optical density (OD) at 600 nm of 0.4. The *A. tumefaciens* suspensions were infiltrated into the leaves of *N. benthamiana* using a needleless syringe. Symptom development was observed visually 5–7 days after infiltration. INF 1 from *P. sojae* was chosen as the positive control (Heese *et al.*, 2007). GFP was used as the negative control.

The purified BxCDP1 protein was diluted in phosphate‐buffered saline (PBS, pH 7.2). To assess the induction of cell death by the recombinant protein produced in *P. pastoris*, solutions of 100 pM to 3 µM purified BxCDP1 protein were infiltrated into *N. benthamiana* leaves; 100 pM to 3 µM purified BSA protein and EV solution were used as negative and blank controls, respectively. The purified BxCDP1 protein (300 nM) was also infiltrated into the leaves of *A. thaliana* ecotype Columbia, tomato (*S. lycopersicum*), and pepper (*C. annuum* var. CM334). The purified BxCDP1 protein (1 µM) was also infiltrated into the leaves of lettuce (*L. sativa* var. *romana*) and wheat (*T. aestivum*). We photographed *N. benthamiana* leaves at 3 days post‐infiltration, *A. thaliana,* and *S. lycopersicum* leaves at 5 days post‐infiltration, and *C. annuum*, *L. sativa,* and *T. aestivum* leaves at 7 days post‐infiltration. The infiltration experiment was performed three times, and three different plants with three inoculated leaves were used for each assay.

### 4.6 Electrolyte leakage assay

Ion leakage from leaf discs of *N. benthamiana* was measured as described previously (Yu *et al.*, 2012). Five *N. benthamiana* leaf discs (9 mm diameter) were collected 7 days after agroinfiltration and floated on 5 ml deionized water for 3 hr with continuous shaking (100 rpm) at room temperature. The initial and final electrolyte leakage values after 30 min of boiling were measured using a conductivity meter (S470 Seven Excellence; Mettler Toledo). Relative electrolyte leakage was calculated by comparing the initial and final values. The experiment was performed three times.

### 4.7 VIGS for* NbBAK1 *and *NbSOBIR1* in *N. benthamiana*

VIGS experiments were conducted using *A. tumefaciens* GV3101 harbouring the pTRV1 vector and pTRV2:*NbBAK1*, pTRV2:*NbSOBIR1/NbSOBIR1‐like*, and pTRV2:*GFP*, as described in previous studies (Wang *et al.*, 2018). At 3–4 weeks post‐infiltration with VIGS constructs, *N. benthamiana* was infiltrated with PVX:*BxCDP1*, PVX:*INF1*, and PVX:*NPP1*. The oomycete PAMP INF1 and effector NPP1 were used as negative and positive controls, respectively. Silenced plants were also infiltrated with 300 nM purified BxCDP1 protein, with EV used as the control. The *N. benthamiana* phenotypes were scored at 5 days post‐infiltration. VIGS effectiveness was assessed according to the phenotype of *PDS*, as described previously (Liu *et al.*, 2002). The infiltration experiment was performed three times, and three different plants with three inoculated leaves were used for each assay.

### 4.8 Sequence analysis

Sequences similar to BxCDP1 were retrieved by querying the BxCDP1 protein against the NCBI protein database using BLASTP (https://blast.ncbi.nlm.nih.gov/Blast.cgi). The signal peptide and transmembrane helices were predicted using the SignalP v. 5.0 server (http://www.cbs.dtu.dk/services/SignalP/) and TMHMM v. 2.0 server (www.cbs.dtu.dk/services/TMHMM/), respectively. BxCDP1 domains were analysed using SMART (http://smart.embl‐heidelberg.de/) (Hu *et al.*, 2019). In addition, the nucleotide sequence of BxCDP1 was queried against the genomes of all other nematodes in the WormBase ParaSite database (https://parasite.wormbase.org/). The *P. haloflavum* protein that most closely matched BxCDP1 was also queried in the genomes of species of closely related genera (*Pararhizobium*, *Rhizobium*, and *Ensifer*) and other microorganisms using local BLAST analysis with a BLAST E‐value cut‐off of 1e−10. Alignment of the amino acid and nucleotide sequences of BxCDP1 and PhCDP1 was performed using BioEdit with ClustalW multiple alignment (Li *et al.*, 2018a).

### 4.9 RT‐qPCR assays

RT‐qPCR assays were carried out using ChamQ SYBR qPCR Master Mix (Low ROX Premixed) (Vazyme) according to the manufacturer's instructions. The silencing efficiencies of *NbBAK1*, *NbSOBIR1*, and its homolog *NbSOBIR1‐like* were validated by RT‐qPCR analysis of cDNA from the leaves of three individual silenced and control *N. benthamiana* plants. At 3 hr post‐infiltration with 300 nM purified BxCDP1 protein, three PTI marker genes (*NbAcre31*, *NbPTI5*, and *NbCyp71D20*) of *N. benthamiana* were normalized against infiltration with EV. The expression levels of the effector at the early stage of host infection were also measured. In addition, relative transcript levels of PR genes (*PtPR‐3*, *PtPR‐4*, and *PtPR‐5*) of *P. thunbergii* were detected when 3‐year‐old seedlings were inoculated with 50 µg/ml purified BxCDP1 protein. The seedlings were inoculated with EV and used as a control. *NbEF1α* of *N. benthamiana*, *Actin* of *B. xylophilus* (GenBank EU100952), and *PtEF1α* were used as constitutively expressed endogenous control genes (Hirao *et al.*, 2012; Ma *et al.*, 2015). All assays were performed three times. Primer sequences are provided in Table S1.

### 4.10 Protein extraction and western blotting

Total protein extraction and immunoblotting were performed according to a previous report (Yin *et al.*, 2013). Briefly, agroinfiltrated *N. benthamiana* leaves were harvested at 2 days post‐inoculation. Total protein extracts were prepared by grinding 400 mg of leaf tissue in 1 ml of radioimmunoprecipitation assay (RIPA) lysis and extraction buffer (Beyotime) in the presence of 0.1 mM phenylmethanesulfonyl fluoride (PMSF) and protease inhibitor cocktail (Beyotime). Total proteins were separated by 12% SDS‐PAGE and transferred to a polyvinylidene fluoride (PVDF) membrane (Bio‐Rad). The membranes were blocked with 5% (wt/vol) nonfat dry milk for 1 hr at room temperature followed by three washes with PBS containing 0.1% Tween‐20. Transient protein expression in *N. benthamiana* was assessed by incubating the membrane with a 1:5,000 dilution of a primary mouse anti‐HA antibody (Abmart) or anti‐RFP antibody (Abcam), followed by incubation with a goat anti‐mouse secondary antibody at a 1:10,000 dilution (IRDye 800, 926‐32210; LI‐COR Biosciences). The purified BxCDP1 protein and EV were analysed by incubating the membrane with a 1:5,000 dilution of a primary rabbit anti‐His polyclonal antibody, followed by incubation with a peroxidase‐conjugated goat anti‐rabbit IgG secondary antibody at a 1:10,000 dilution (Zhongshan Bio‐technique). The proteins were visualized using an Odyssey LI‐COR imaging system. Equal protein loading was confirmed by Ponceau S staining.

### 4.11 Measurement of ROS

Measurement of ROS was performed according to a previous report (Yin *et al* *.*, 2013). ROS production was monitored by a luminol/peroxidase‐based assay using leaf discs (0.5 cm diameter) collected from 5‐week‐old *N. benthamiana* plants and floated overnight in 200 μl of sterile H_2_O in a 96‐well plate. H_2_O was replaced with luminol (35.4 μg/ml)/peroxidase (10 μg/ml) reaction solution in EV, 100 nM flg22 (GenScript Biotech Corporation), and 1 μM purified BxCDP1 protein. Luminescence was measured using a GLOMAX96 microplate luminometer (Promega).

### 4.12 Inoculation assay and *P. thunbergii* cell morphology observation

Three‐year‐old *P. thunbergii* seedlings were inoculated with 1 ml purified BxCDP1 protein (final concentration 50 μg/ml) and EV as described previously (Zhong *et al.*, 2013). At 4 hr post‐inoculation, approximately 2,000 nematodes (a mixture of juveniles and adults) were inoculated into these seedlings. The levels of *B. xylophilus* infection were tested, and the morbidity degrees of *P. thunbergii* seedlings were recorded. The infection assay of *B. xylophilus* was performed three times.

Meanwhile, at 10 days post‐inoculation with purified BxCDP1 protein and EV, c.3 mm of seedling stem was collected from 1 cm below the inoculation site, and transversal 1‐mm thick sections were cut using a surgical blade. The specimens were processed as follows:

- Double fixation: The specimens were fixed with 2.5% glutaraldehyde in phosphate buffer (PB, pH 7.0) for at least 4 hr, washed three times in PB, postfixed with 1% OsO_4_ for 1 hr and washed three times in the PB.
- Dehydration: The specimens were dehydrated in a graded series of ethanol (30%, 50%, 70%, 80%, 90%, 95%, and 100%) for approximately 15–20 min at each step and transferred to absolute acetone for 20 min.
- Infiltration: The specimens were placed in a 1:1 mixture of absolute acetone and the final Spurr resin mixture for 1 hr at room temperature and transferred to a 1:3 mixture of absolute acetone and the final resin mixture for 3 hr and then to the final Spurr resin mixture overnight.
- Embedding and ultrathin sectioning: Specimens were placed in capsules containing embedding medium and heated at 70 °C for approximately 9 hr. The sections were stained with uranyl acetate and alkaline lead citrate for 15 min and observed by TEM (JEM‐1400).

## AUTHOR CONTRIBUTIONS

Xiao‐Qin Wu and Long‐Jiao Hu were the leading investigators of this research programme. Long‐Jiao Hu, Xiao‐Qin Wu, and Hai‐Yang Li planned and designed the research; Long‐Jiao Hu performed the majority of experiments with the help of Xin Huang and Yu Li; Xiao‐Qin Wu and Yuan‐Chao Wang contributed reagents, materials, and analysis tools; Long‐Jiao Hu and Hai‐Yang Li analysed the data; Long‐Jiao Hu wrote the paper with suggestions from Xiao‐Qin Wu, Yuan‐Chao Wang, and Yan Wang. All authors commented on the article before submission.

## Data Availability

The data used to support the findings of this study are available from the corresponding author upon request.
