# Supplementary figures and images for: BxCDP1 from the pine wood nematode Bursaphelenchus xylophilus is recognized as a novel molecular pattern
Source: Mol Plant Pathol. 2020 Apr 21;21(7):923–35. doi: 10.1111/mpp.12939 (PMC7280032; doi:10.1111/mpp.12939)

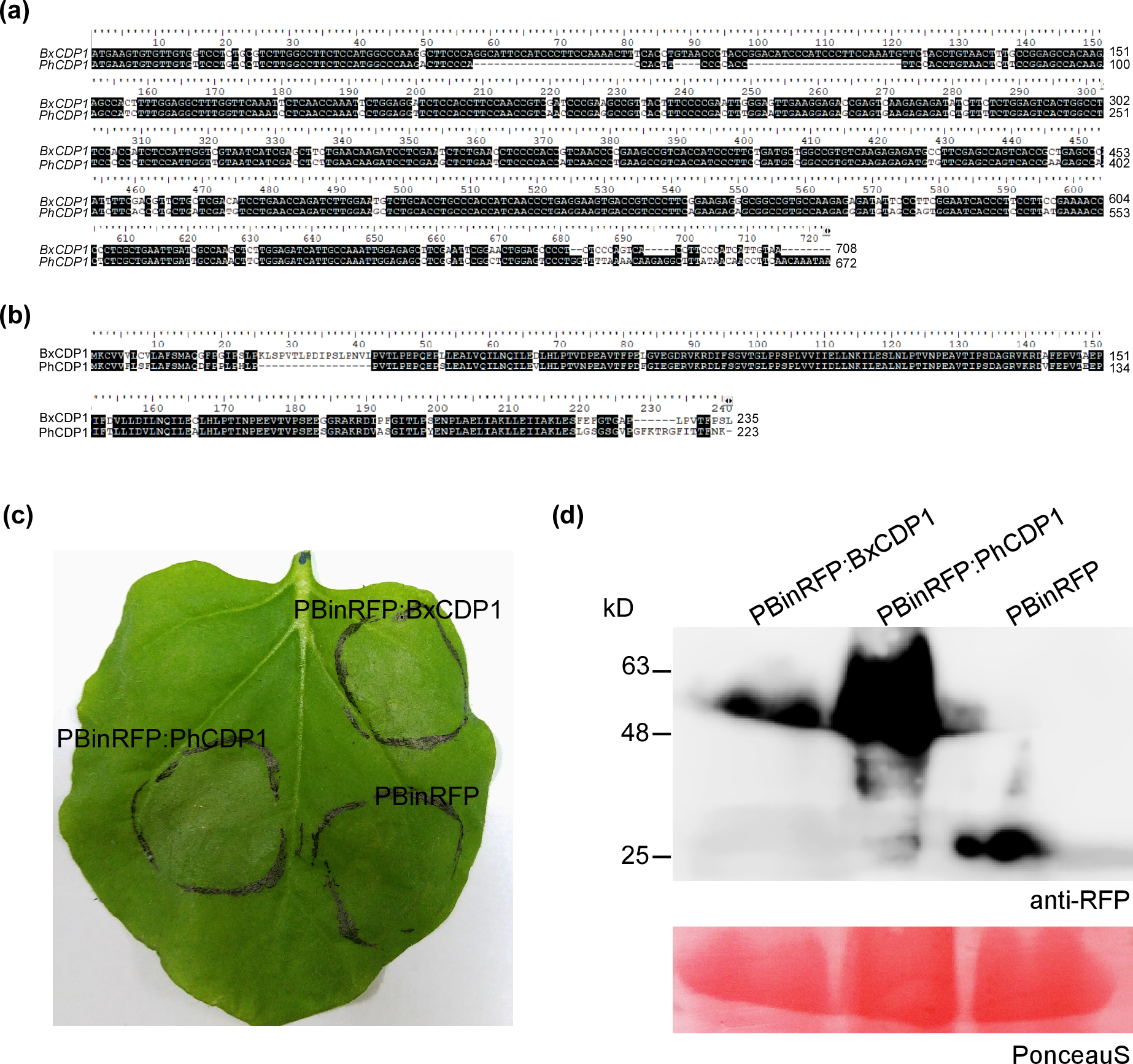

Supplement: Supplementary file 1 [file MPP-21-923-s001.tif]

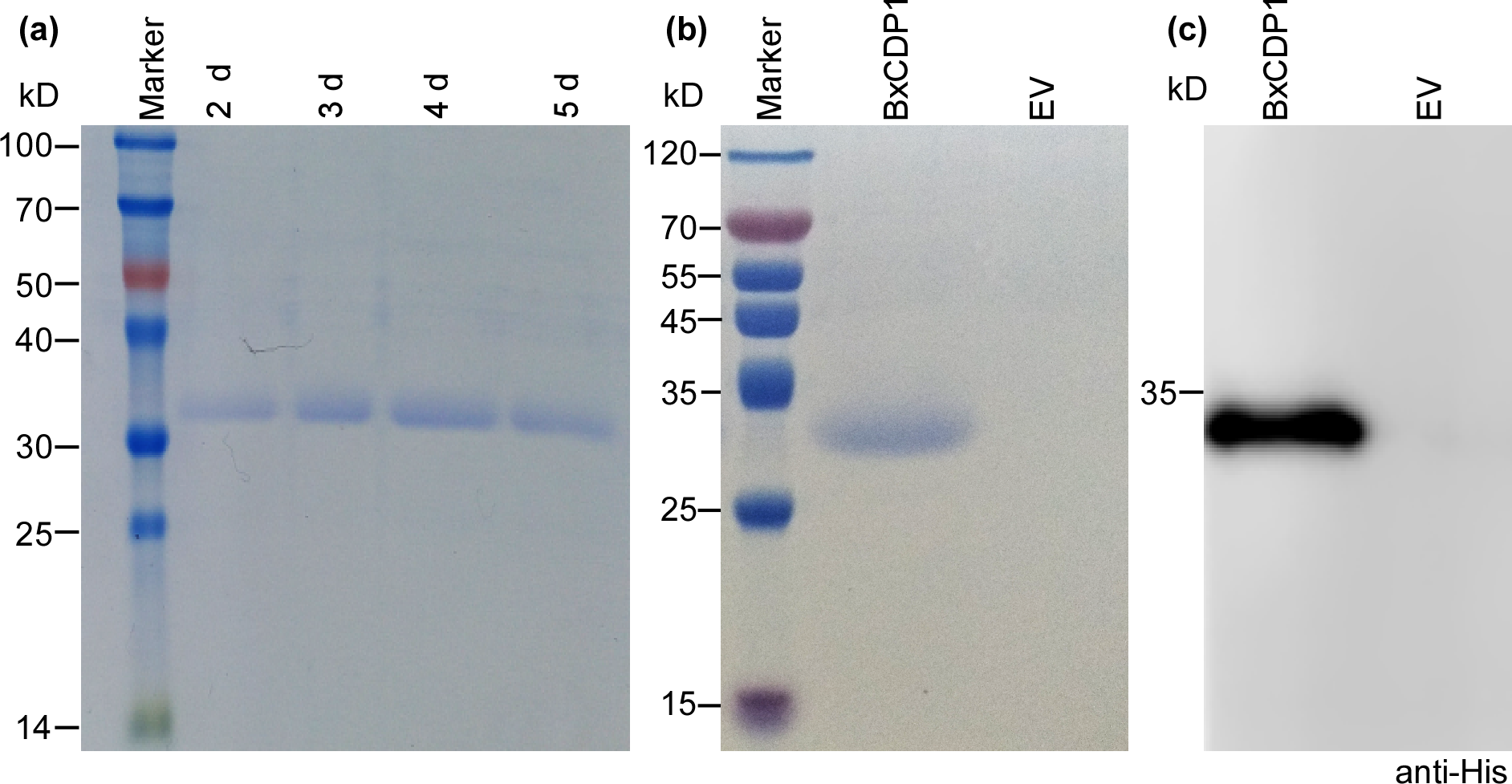

Supplement: Supplementary file 2 [file MPP-21-923-s002.tif]

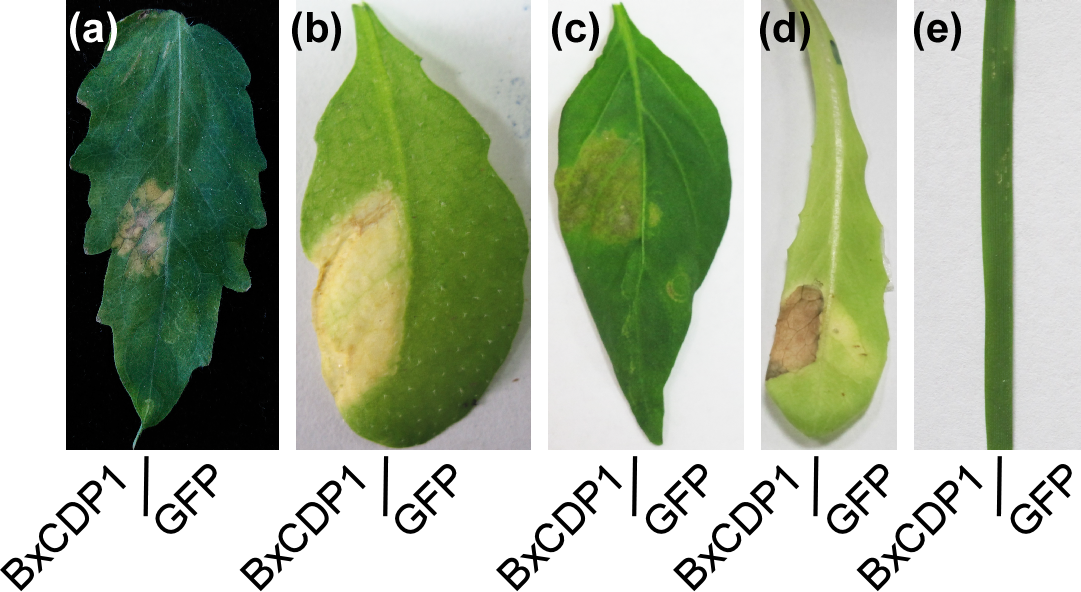

Supplement: Supplementary file 3 [file MPP-21-923-s003.tif]

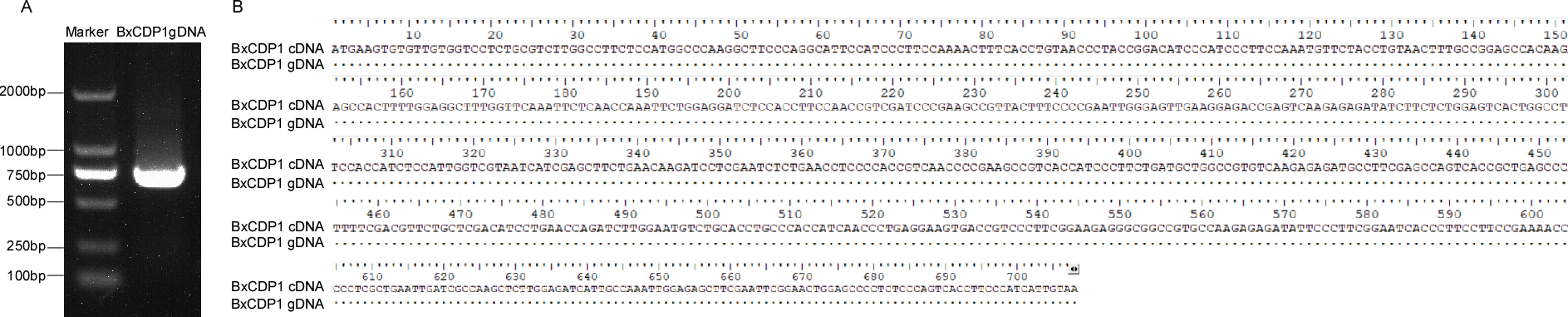

Supplement: Supplementary file 4 [file MPP-21-923-s004.tif]
